# Management of Distal Radius Fractures: Comparison of Three Methods

**DOI:** 10.7759/cureus.9875

**Published:** 2020-08-19

**Authors:** Cenk Ermutlu, Murat Mert, Emrah Kovalak, Enes Kanay, Abdullah Obut, Yusuf Öztürkmen

**Affiliations:** 1 Orthopaedics, Bursa Uludag University School of Medicine, Bursa, TUR; 2 Orthopaedics and Traumatology, Yeni Yüzyıl Üniversitesi, İstanbul, TUR; 3 Orthopaedics and Traumatology, Biruni University Medical School, Istanbul, TUR; 4 Orthopaedics and Traumatology, Istanbul Beykoz State Hospital, Istanbul, TUR; 5 Orthopaedics and Traumatology, Bursa State Hospital, Bursa, TUR; 6 Orthopaedics and Traumatology, Istanbul Training and Research Hospital, Istanbul, TUR

**Keywords:** distal radius fracture, external fixator, k-wire, pinning, plate fixation, wrist fractures

## Abstract

Introduction

Distal radius fractures are the most common type of all extremity fractures. It is generally accepted that fractures with more than 2 mm step-off in the radiocarpal joint and greater than 10 degrees dorsal tilt should be treated surgically. However, the ideal technique for surgical management is still a point of debate. We performed cross-sectional data analysis to compare the results of three treatments methods - volar locking plate (VLP), external fixation (EF), Kirschner wire (K-wire) - in patients with distal radius fractures, and compared the clinical, functional, and radiological results

Materials and methods

Forty-four patients with distal radius fractures who underwent fixation with VLP, K-wire or EF between 2011 and 2013 were included in the study. All fractures were classified according to the Müller’s Arbeitsgemeinschaft für Osteosynthesefragen (AO) and Frykman's classifications. Routine radiographs were taken at the postoperative three weeks, six weeks, and three months. Radial inclination, volar tilt, radial length and ulnar variance were assessed on the follow-up visits and additionally at the follow -up for the study. The patient-based Disabilities of the Arm, Shoulder and Hand (DASH) score system and the physician-based MAYO scale were used to evaluate functional outcomes. Radiological and functional outcomes between three surgical modalities were compared and statistically analyzed.

Results

The average age at the time of surgery was 52 years (range = 35-69 years). Of a total of 44 patients, 28 were operated with VLP, 11 were with K-wire and five with EF. Satisfactory reduction was achieved in all fractures, and all of the fractures healed. DASH and MAYO scores were similar in all groups. Regarding radiographic parameters, there was no significant difference in radial inclination, volar tilt, radial length and ulnar variance between the treatment modality groups. When evaluated based on fracture geometry, the DASH score was significantly higher in the patients with AO23A type fracture compared to the patients with AO23B and AO23C type fractures. As for MAYO score, all AO23 groups had similar outcomes.

Conclusions

Surgical treatment options VLP, EF, and K-wire provide adequate fixation, satisfactory radiological, and functional results for the management of distal radius fractures of various severities. The optimal treatment approach depends on individual features, and the choice for an internal fixation or closed reduction method for the restoration of wrist function should be evaluated thoroughly by the operating surgeon considering the patient-related variations.

## Introduction

Distal radius fractures are the most common type of all extremity fractures. Although high-energy distal radius fractures are more frequent in younger people, low energy fractures in the elderly may still require reduction [1]. Restoration of wrist function and maintaining the radiocarpal and radioulnar joint mechanics at the maximum obtainable level are of utmost concern; deciding between non-operative or operative management is an essential task for orthopaedic surgeons. Articular malalignment, loss of reduction, and inadequate fixation can result in posttraumatic osteoarthrosis, shortening at the fracture site, and impaired wrist and hand function [2].

Distal radius practice guidelines of American Academy of Orthopaedic Surgeons recommend with moderate strength that, fractures with post-reduction radial shortening >3 mm, dorsal tilt >10 degrees, or intra-articular displacement or step-off >2 mm should be treated surgically rather than cast fixation [3]. 2018 Best Practice for the Management of Distal Radial Fractures by British Orthopaedic Association and British Society for Surgery of the Hand proposes that surgical intervention is indicated if a 4-5 mm positive ulnar variance is present for patients between 38 and 58 years of age [4].

Percutaneous pinning with Kirschner wire (K-wire), volar locking plate (VLP) and external fixation (EF) are among the fixation techniques used in the clinical practice for the treatment of distal radial fractures. Although there are various reports claiming the superiority of one method over another, the decision on the treatment modality is multifactorial. The patient's age, occupation, familiarity of the procedure to the surgeon, the comorbidities such as tendon and median nerve injuries should be taken into account, as well as the fracture configuration [5-7]. Internal fixation with VLP is the most commonly used treatment for unstable distal radius fractures despite its relatively higher complication rate due to deep dissection of soft tissue around the fracture region, and the need for a removal surgery for intra-articular fracture cases [8]. On the other hand, closed reduction techniques EF and K-wire have the advantages of being less invasive with easy application and lower costs [9].

We performed cross-sectional data analysis to compare the results of three treatments methods - VLP, EF, K-wire - in patients with distal radius fractures, and compared the clinical, functional, and radiological results in an attempt to determine which modality provides better outcome and satisfactory restoration of the wrist function.

## Materials and methods

Fourty-four patients with distal radius fractures who underwent fixation with VLP, K-wire or EF between 2011 and 2013 were included in the study. Hospital information system medical records and radiology archiving system were used to obtain the patient data. Inclusion criteria were as follows: age of 18 years and older, definite diagnosis of distal radius fracture by radiograph or CT scanning, no prior surgery at the injured wrist region, unilateral fracture, treatment with VLP, K-wire fixation or EF within one week. Exclusion criteria were fractures treated after a week, pathological fractures due to chronic bone-related diseases or tumors, and concomitant fractures at the injured limb.

All fractures were classified according to the Müller’s Arbeitsgemeinschaft für Osteosynthesefragen (AO) and Frykman's classifications. According to the AO classification, there were two A2, four A3, eight B1, five B2, nine B3, eight C1, seven C2, and one C3 fractures. While the fractures were sub-grouped according to Frykman's classification, there were three Type 1, three Type 2, twelve Type 3, nine Type 4, one Type 6, seven Type 7, and nine Type 8 fractures. Surgical indications were determined according to current guidelines by the consensus of the operating surgeons, and the most appropriate treatment method was determined for each patient.

Surgical techniques

An initial reduction maneuver was attempted before each treatment method. Procedures were performed under general anesthesia with patient in supine position under fluoroscopic guidance.

VLP fixation

The fracture was fixed using the volar approach through the interval between the flexor carpi radialis and radial artery. MISS distal radius plates (TST Tıbbi Aletler San ve Tic Ltd. Şti., Istanbul) with locking screws were used.

External fixation

An external fixator (Dynamic Angled Clamp Wrist Fixator, TST Tıbbi Aletler San ve Tic Ltd. Şti., Istanbul) was fixed on the radius with 3 or 4-mm Schanz pins according to the surgeons’ preference. Initially, two Schanz screws were placed onto the radius diaphysis under fluoroscopic guidance, followed by two Schanz screws onto the 2nd metacarp. The wrist was kept on flexed position during the placement of the screws to prevent contracture due to blockade of the tendons by screws. The fracture was reduced and the system secured. Additional percutaneous 2 mm K-wires were inserted under fluoroscopy in order to secure the wrist integrity when necessary.

Percutaneous pinning (K-wire)

After the reduction of the fracture under fluoroscopic guidance, two or more - according to the necessity - 2 mm or 1.5 mm K- wires were placed medially and laterally. Stability was checked and a short arm splint was applied.

None of the patients required autogenous bone graft or allograft. Radiologic parameters (radial inclination, volar tilt, radial length, ulnar variance) were evaluated following all procedures. Operations were performed by five different surgeons with high levels of surgical expertise. Active and passive exercise of the digits and the elbow were initiated on the first postoperative day. Wound dressing was changed once daily, and when necessary in the early postoperative time. In groups of VLP and external fixation no splint was applied. Splints were only applied in percutaneous pinning group for four weeks. The external fixator and K-wires were removed in six (6-8) weeks after surgery. Routine radiographs were taken at the postoperative three weeks, six weeks, and three months. Radial inclination, volar tilt, radial length and ulnar variance were assessed on the follow-up visits and additionally at the follow -up for the study.

Follow-up and postoperative evaluation

The patient-based DASH score system and the physician-based MAYO scale were used to evaluate functional outcomes. DASH questionnaire was validated by the patient evaluating the ability to perform the daily activities. MAYO scale was validated by the orthopaedic surgeon based on the pain, functional status, range of wrist motion and grip strength status. Standard posteroanterior and lateral radiographs were used to measure radiologic parameters.

Statistical analysis

Statistical Package for the Social Sciences (SPSS), version 15.0 for Windows (SPSS Inc., Chicago, IL) program was used for statistical analysis. Descriptive statistics were given in number and percentage for categorical variables, mean and standard deviation for numerical variables. Comparisons between the two independent groups were made by Student’s t test or Mann-Whitney U test, based on their data distribution status. Independent numerical variables more than two group comparisons were made with one-way ANOVA or Kruskal Wallis test, based on their data distribution status. Subgroup analyzes were performed with parametric test Tukey test, nonparametric test with Mann Whitney U test, and interpreted with Bonferroni correction. The ratio of categorical variables between the groups was tested by Chi-square analysis with Monte Carlo simulation when necessary. The relationship between numerical variables was tested by Spearman correlation analysis. Statistical significance level was accepted as p <0.05.

## Results

Twenty-five patients were men, and 19 were women. The average age at the time of surgery was 52 years (range = 35-69 years). The mean follow-up period was 18 (14-24) months. The mean age was 49.9±14.1 years in the VLP group, 52.3±12.3 in the K-wire group and 55.0±11.0 in the EF group. Twenty-one patients had a fracture of the right radius and 23 patients had a fracture of the left radius. Out of the 20 patients with dominant hand data, dominant hand was affected in nine patients. Of a total of 44 patients, 28 were operated with VLP, 11 were with K-wire and five with EF. The mean follow-up was 13.6±11.3 months for the VLP group; 10.6±9.0 months for the K-wire group and 6.2±5.2 months for the EF group. There was no significant difference between the fixation methods in terms of age, gender, side of injured wrist, AO or FRYKMAN classification and duration of follow-up (Table 1).

**Table 1:** Descriptive comparison of perioperative variables between the surgical management groups

Variable	Volar locking plate (n=28) (n, % or mean ± SD )	K-wire (n=11) (n, % or mean ± SD )	External fixator (n=5) (n, % or mean ± SD )	p value
Age	49.9± 14.1	52.3± 12.3	55.0± 11.0	0.690
Gender				
Female	11 (39.2)	4 (36.3)	2 (40.0)	0.820
Male	17 (60.8)	7 (63.7)	3 (60.0)	
Wrist fractured				
Right	15 (53.6)	4 (36.4)	2 (40.0)	0.607
Left	13 (46.4)	7 (63.6)	3 (60.0)	
Dominant hand				
Yes	8 (28.6)	1 (9.0)	0 (0.0)	0.069
No	4 (14.2)	5 (40.5)	2 (40.0)	
Missing	16 (57.2)	5 (40.5)	3 (60.0)	
AO23 classification of the fracture				
A2	1 (3.6)	1 (9.1)	0 (0.0)	0.149
A3	4 (14.3)	0 (0.0)	0 (0.0)	
B1	5 (17.9)	3 (27.3	0 (0.0)	
B2	4 (14.3)	1 (9.1)	0 (0.0)	
B3	8 (28.6)	1 (9.1)	0 (0.0)	
C1	4 (14.3)	2 (18.2)	2 (40.0)	
C2	2 (7.1)	3 (27.3)	2 (40.0)	
C3	0 (0.0)	0 (0.0)	1 (20.0)	
Frykman classification of the fracture				
1	2 (7.1)	1 (9.1)	0 (0.0)	0.176
2	2 (7.1)	0 (0.0)	1 (20.0)	
3	10 (35.7)	2 (18.2)	0 (0.0)	
4	7 (25.0)	2 (18.2)	0 (0.0)	
6	1 (3.6)	0 (0.0)	0 (0.0)	
7	2 (7.1)	4 (36.4)	1 (20.0)	
8	4 (14.3)	2 (18.2)	3 (60.0)	
Follow-up duration	13.6±11.3	10.6±9.0	6.2±5.2	0.331

Satisfactory reduction was achieved in all fractures, and all of the fractures healed in our study group. None of the patients experienced surgical site infection. None of the patients required a revision at the operated wrist. DASH and MAYO scores were similar in all groups. Evaluation of postoperative radiographs showed that the average radial height was 7.4±7.6 mm, 12.4±4.8 mm and 8.6±7.1 mm, whereas volar tilt was 6.7±6.6°, 6.4±5.5° and 6.0±5.6°; along with radial inclinations of 20.4±4.1°, 22.1±4.4°, and 21.4±8.2° in the VLP, K-wire, and EF groups, respectively. Regarding radiographic parameters, there was no significant difference in radial inclination, volar tilt, radial length and ulnar variance between the treatment modality groups (Table 2).

**Table 2:** Comparison of radiologic parameters and functional outcomes between the surgical treatment groups at the last visit

Variable	Volar locking plate (n=28)	K-wire (n=11)	External fixator (n=5)	p value
Radial inclination (degr)	20.4±4.1	22.1±4.4	21.4±8.2	0.573
Volar tilt (degr)	6.7±6.6	6.4±5.5	6.0±5.6	0.903
Radial height (mm)	7.4±7.6	12.4±4.8	8.6±7.1	0.146
Ulnar variance				
Positive	2 (7.1)	3 (27.3)	1 (20.0)	0.444
Negative	16 (57.1)	6 (54.5)	3 (60.0)	
Normal	10 (35.7)	2 (18.2)	1 (20.0)	
Mayo score (points)	85.7±14.4	85.7±13.4	72.5±3.6	0.346
DASH score (points)	16.0±23.5	22.3±18.5	25.1±30.6	0.273
DASH score during activity (points)	12.5±22.4	22.9±39.7	43.8	0.452

The results indicated that all treatment methods provided adequate stability. There was significant negative correlation between the MAYO score and age, follow-up period and volar tilt, and a positive correlation between the follow-up period and radial length in the patient group (r=-0,656; -0,465; 0,502, respectively; p < 0.001 for all correlations) (Table 3).

**Table 3:** Correlation of peri- and postoperative variables and radiological and functional measures between the surgical treatment groups at the last visit DASH, Disabilities of the Arm, Shoulder and Hand

	Age		Follow-up duration	
	rho	p	rho	p
Radial inclination (degr)	0.019	0.904	0.002	0.989
Volar tilt (degr)	-0.078	0.615	-0.465	0.001
Radial height (mm)	0.010	0.951	0.502	0.001
MAYO score (points)	-0.656	0.001	0.363	0.081
DASH score (points)	0.265	0.245	-0.338	0.134
DASH score during activity (points)	0.024	0.931	-0.237	0.394

The DASH score was significantly higher in the patients with AO23A type fracture compared to the patients with AO23B and AO23C type fractures. As for MAYO score, all AO23 groups had similar outcome (Table 4).

**Table 4:** Relationship between fracture types according to AO23 classification and functional outcomes

		DASH score (points)		MAYO score (points)	
		Mean±SD	Median	Mean±SD	Median
AO23	A	42.4±36.5	50.8	70.0±13.2	65
	B	6.0±4.7	5	88.3±13.5	95
	C	19.6±18.8	13.95	85.4±12.5	90
	p	0.048		0.125	

## Discussion

Distal radius fractures are among the most common extremity fractures affecting both young and elderly individuals. Restoration of the wrist function to pre-injury levels is of primary concern for the orthopaedic surgeons due to the involvement of the joint in a wide variety of daily activities. Treatment options are based on the type and characteristics of the injury and the radiological findings, and either an open or closed surgical treatment may be required if the initial reduction is not in acceptable limits [10]. Furthermore, the functional outcomes with conservative treatment are poor, especially in younger patients with intra-articular fractures [11]. The operative technique should be carefully selected to achieve a successful treatment outcome and to preserve the activity level, bone quality, and general life quality of the patient. The present study was designed to evaluate the radiological and functional results of operative management of distal radial fractures using VLP, external fixation, and K-wire.

Leung et al. reported that the VLPs are superior to EF for the treatment of intraarticular fractures [12]. In our study, we successfully treated intraarticular AO23C and Frykman type VII and VIII fractures using three different techniques, and did not find a significant difference in functional and radiological outcome. Although we did not use EF for the treatment of intraarticular Frykman type III and IV fractures, this might be a result of a relatively small number of patients in this group.

Yu et al. compared the VLP and EF for the treatment of type AO23C2/C3 distal radius fractures and concluded that VLP is superior to EF in maintaining joint stability and wrist mobility [13]. They noted that the functional DASH score was similar between these treatment groups, which is in concordance with our findings. The average postoperative radial inclination, volar tilt, radial length, and the ratio of ulnar variance did not differ between the treatment modalities. The functional scores of our study patients did not differ between the treatment types, and the patients had excellent to good functional results.

Meta-analyses report that VLP is superior to EF and K-wire in terms of ulnar variance, for all types of radius fractures [14,15]. The ratio of patients with ulnar variance (both positive and negative) was similar in our study groups for all treatment methods. Young Hak Roh et al. reported that VLP was superior to EF in terms of ulnar variance for the patients with AO-type C2 and C3 fractures [16]. In contrast with their findings, while we constituted a subgroup of patients with AO23C type fractures, radiological findings did not differ between the treatment methods. We conclude that the differences might be resulting from the variations in study design, patient characteristics, data collection, and duration of the follow-up period. However, the similar radiological results with respect to postoperative inclination, volar tilt, and radial length between the treatment modalities are in accordance with the previous reports [9,17]. However, it should be noted that radiological and functional findings might not be correlated for all patients with distal radius fractures treated with different surgical management methods. Thus, long-term follow-up durations might yield similar functional results even though radiological evaluation parameters differ between the treatment groups. Although Gravier et al. reported better ulnar variance results with additional pins, while they used extra intra- and extra-focal pins, however, it should be noted that this might increase the risk of the ratio of pin-related complications [18]. Thus, the risk for any possible hazards should be taken into consideration, and avoiding measures should be taken by the operating surgeon.

The complication rates after surgical management of distal radius fractures vary in different studies depending on the injury severity, involvement of the joint, and the treatment method [19,20]. Yu et al. reported a 34.5% and 28.2% complication rate in their patients treated with EF and VLP, respectively [13]. The most common complications for surgical management of distal radius fractures include loss of reduction, infection, joint stiffness due to nerve injury, tendon rupture, osteoarthritis, and tendonitis [8,21,22].

VLP method has numerous functional and structural advantages, including its anatomically shaped design, early mobilization, and well-preserved wrist function. Several reports show that the VLP is related to tendon related complications, even necessitating plate removal and resurgery [8].However, we did not experience such complications in our patient group, possibly as a result of appropriate patient selection, careful soft tissue dissection and precise screw lengths. Farhan et al. reported in their series of patients with AO23C3 distal radius fractures that 16% of the patients required plate removal [23]. In our series, we had only one patient with an AO23C3 type fracture, and we treated the patient using EF, and the patient had an event-free follow-up period. However, we had six patients with AO23C type fractures treated with VLP fixation, and none of the patients required removal of the plate.

In a study by Yu et al., the ratio of additional K-wires for EF augmentation was 70%, with a 30% ratio of fractures requiring bone graft [13]. Seitz et al. reported that the EF with additional K-wires resulted in a satisfactory result in 92% of the patients [24]. Since we used one to two additional percutaneous K-wires in 40% of the patients treated with EF in order to increase the stability, we obtained satisfactory radiological and functional results, and did not experience any pin tract infection and injury of the sensory branch of the radial nerve in our patients. However, the small sample size of this group (5/44) might not be adequate to have a conclusion in this matter. Postoperative care as the use of oral antibiotics and intensive pin tract care, as well as intraoperative measures, are of crucial importance for the prevention of pin-related complications. There was no incidence of posttraumatic arthritis reported in our patient population, possibly due to the absence of a long-term follow-up. Although it did not reach statistical significance, the relatively higher age in the EF and K-wire group is due to the common implication of closed reduction methods in elderly individuals and osteoporotic patients for the reason that screw insertion into the osteoporotic bone tissue is challenging and prone to failure and loosening.

There were several limitations to this study. Firstly, this is a retrospective, nonrandomized, comparative trial with a small sample size and with the heterogeneity of the treatment groups. Secondly, the procedures in this study were performed by different surgeons of various experience levels, which might affect the outcomes. Thirdly, the demographic variables and reports from the physical treatment clinic are not present, so it is not possible to evaluate the lifestyle, occupational risks, adherence to physical therapy for the patients. However, the number of variables and different functional scoring systems assessed are the most substantial aspects of this study.

## Conclusions

Surgical treatment options VLP, EF, and K-wire provide adequate fixation, satisfactory radiological, and functional results for the management of distal radius fractures of various severities. The fracture configuration is the main factor in determining functional outcomes. On the contrary to the expectation, fractures without articular involvement have worse disability scores following treatment. The optimal treatment approach depends on individual features, and the choice for an internal fixation or closed reduction method for the restoration of wrist function should be evaluated thoroughly by the operating surgeon considering the patient-related variations.
